# Perspectives on program duration and research: A survey of graduates of a 3‐year medical physics residency program

**DOI:** 10.1002/acm2.14534

**Published:** 2024-10-15

**Authors:** Martina Hurwitz, David P. Gierga, Brian Winey, Piotr Zygmanski, W. S. Kiger, Yulia Lyatskaya

**Affiliations:** ^1^ Department of Radiation Oncology Beth Israel Deaconess Center Boston Massachusetts USA; ^2^ Harvard Medical School Boston Massachusetts USA; ^3^ Department of Radiation Oncology Massachusetts General Hospital Boston Massachusetts USA; ^4^ Department of Radiation Oncology Brigham and Women's Hospital Boston Massachusetts USA

**Keywords:** medical physics, medical physics residency, training

## Abstract

**Purpose:**

The goal of this manuscript is to evaluate strengths and weaknesses of a 3‐year medical physics residency program with the first year dedicated to research and the remaining 2 years dedicated to clinical training.

**Methods:**

An anonymous survey was distributed to graduates of a 3‐year medical physics residency program with a dedicated year of research. Questions focused on several categories: (1) in retrospect, factors graduates considered at the time of application, (2) aspects of respondents’ career and life after graduating from residency, (3) respondents’ opinions on the residency duration, and (4) research productivity during residency.

**Results:**

Of 22 graduates who were contacted, 17 filled out the survey. In retrospect, the most impactful consideration at the time of application was quality of clinical training. Extra time for research was reported as the factor with the most positive career impact and financial considerations as the most negative impact of completing a 3‐year residency. Preference for a 3‐year residency at the time of application and in retrospect was expressed by 47% and 88% of respondents, respectively. 71% of respondents would recommend applying to a 3‐year program to current residency candidates, and 29% respondents said their recommendation on entering a 3‐year program would depend on the candidate's interests. 76% of respondents preferred dedicated time for research during residency.

**Conclusions:**

The optimal duration of medical physics residency depends on the goals and career objectives of the incoming residents. Two years may be an optimal duration for clinical training. For candidates interested in a career with a substantial research component, a 3‐year program may be a good option, and graduates express favorable opinions after completing such a program. A 4‐year residency duration is not viewed favorably by the graduates.

## 1 INTRODUCTION

Medical physicists play a crucial role in radiation oncology departments, working alongside physicians, radiation therapists, and other medical personnel to design and deliver radiation treatments, bring innovative technology to the clinic, and ensure ongoing quality and safety. Educational requirements for becoming a medical physicist include earning a graduate degree in physics or a related field and completion of a medical physics residency.^1^, ^2^ In addition, certification by the American Board of Radiology (ABR)^3^ is typically required for independent work or solo practice. As the radiation oncology field develops and new technologies are introduced,^4^, ^5^, ^6^ medical physics education is adjusted for modern needs.^7^, ^8^, ^9^, ^10^ Multiple documents exist describing education requirements for medical physics residencies including graduate curriculum requirements,^11^, ^12^, ^13^ Commission on Accreditation of Medical Physics Education Programs (CAMPEP) standards,^14^ and The American Association of Physicists in Medicine (AAPM) guidelines.^15^, ^16^ While the main focus of these documents is on clinical training, it is also recognized that medical physics as a profession is multifaceted and includes clinical service and consultation, research and development, and teaching.^17^

Many medical physicists conduct research throughout their careers, driving innovations and improving treatment quality. While residency programs must focus primarily on clinical training, providing research opportunities during residency may be beneficial both to residents and to the larger field of medical physics. At the time of residency, physicists may be at a productive stage in their research career and could be well poised to continue existing research projects or work on projects of interest at their residency institutions. During residency, physicists may receive valuable research training with mentors at their residency institutions, creating a foundation for becoming future independent research group leaders. Research may also be a way to learn more about medical physics as a whole, and publishing papers will help their post‐residency job search. Currently, no standards exist for incorporating research into residency training, and residency programs have taken different approaches to give residents the opportunity to do research, including adding dedicated research time to the 2 years of required clinical training. It is of interest to evaluate whether an additional year of residency training dedicated to research has provided a positive framework for research during residency, and whether it has been helpful to achieving the goals of residents.

Including a 3rd year of residency training to provide time for research may be attractive to those physicists following an “alternative path” into medical physics careers.^16^, ^18^ Physicists following this path must complete the full didactic curriculum required by CAMPEP, which is not possible during a 2‐year residency, but can be done during a 3‐year residency. In addition, the added year dedicated to research may provide physicists following this path an opportunity to build an understanding of medical physics through their research projects.

However, extending the duration of residency may have drawbacks as well. Some residents may not want or need to do research during these years of their career. In addition, extending the duration of a residency program means that residents spend more time in a temporary employment situation with a relatively low salary.

In this report, we evaluate the preferred residency duration and the benefits and challenges of a 3‐year medical physics residency program with a year dedicated to research as viewed by the graduates of such a program. The program was specifically designed to provide incoming residents the opportunities for research during their residency and the ability to fulfill the course requirements in the CAMPEP curriculum.^19^

We believe that this report will provide valuable insight to medical physics educators about the optimal length of residency training with a research component, the benefits and drawbacks of this duration, and the appropriate time allocation for clinical training and research. It may help other residencies decide if a 3‐year program is an attractive option for their institution. It will also help prospective applicants in considering various residency choices.

## 2 METHODS AND MATERIALS

This report focuses on the perceptions of graduates of a specific residency program (the Harvard Medical Physics Residency Program). This residency is a 3‐year program structured to allow residents to focus on research during their 1st year, followed by 2 years of clinical training rotations. The structure and management of the residency is described elsewhere.^19^ Residents are required to have a Ph.D., although their degree does not have to be from a CAMPEP‐accredited program. The residency has participated in the MedPhys match since 2015.

During the dedicated research year, residents work on a particular project with a faculty research mentor. Projects are designed by the research mentor with a scope that will allow the resident to submit at least one first‐author publication based on this year of research. Incoming residents are offered a variety of projects on different topics and can rank them according to their interests. Research topics are not necessarily clinically oriented and are often part of larger funded research efforts. The mentor: resident ratio is 1:1, though there may be other research trainees in the same group and residents frequently work together with students and post‐doctoral fellows during their research year.

Residents are sheltered from clinical expectations during the dedicated research year but have the opportunity to take classes in a CAMPEP‐accredited certificate program. This opportunity to take classes during a non‐clinical year means that residents can take all courses required by CAMPEP during the residency program.

A survey was distributed to graduates of the residency program with a free online format (Google Forms). The goal of the survey was to collect perceptions of graduates of various aspects of the residency program, particularly its duration and structure, and to understand whether changes to the structure were desired. At the date of the survey distribution, the residency program had 23 graduates. Out of these graduates, one graduate did not have a known email address. The other 22 graduates were contacted and asked to fill out the survey. Responses were anonymous.

In total, there were 21 questions on the survey. The questions focused on several categories and are paraphrased below:

1. Information about the respondents:
  - Years since graduation
  - What CAMPEP‐accredited degrees, if any, they held when starting the residency
2. In retrospect, factors considered at the time of residency application:
  - The impact of various factors of the residency program (the ability to complete all CAMPEP‐required coursework, a year dedicated to research, the quality of clinical training, opportunities for leadership development, and work/life balance) on their decision to apply
  - At the time of application, their preference for the duration of the residency
3. Career and life aspects affected by graduating from a 3‐year program
  - Perceived benefit to their career of various aspects of a 3‐year residency program (extra time for research, extra time for classes, extra time for clinical training, and others)
  - How graduating from a 3‐year program affected various post‐residency factors in their lives and careers (finding a job immediately after residency, finding a job that fulfills their career goals, being promoted, and others)
  - Whether they currently hold clinical, academic, or research leadership positions
  - Whether the respondent is satisfied with the way their time is currently divided into clinical, research, and other academic pursuits
  - How many years of experience from residency they were given credit for in salary negotiations
4. Opinions on the residency duration
  - In retrospect, the number of years they would ideally have spent in residency
  - Would they recommend to current candidates to apply to 3‐year programs
  - Should there be 2 years of clinical training during residency
    - If not, how many should there be?
  - Their preference for dedicated time for research during residency
    - If this was their preference, what year within the residency would be best for dedicated research
5. Research productivity during residency:
  - How many papers respondent submitted in the following categories:
    - During residency, based on the project during the dedicated research year
    - During residency, based on other work
    - After residency, based on the project during the dedicated research year
6. Any other comments

The majority of questions were multiple‐choice; responses to these questions were analyzed as aggregates or percentages of total answers and presented in pie charts or tables. For questions 2a, 3a, and 3b listed above, respondents were asked to rank several factors on a scale of 1 (most negative) to 5 (most positive). These ranked questions were analyzed by calculating the average and standard deviation of rankings over all respondents for each factor. Blank or N/A responses were not taken into account in the analysis.

## 3 RESULTS

### 3.1 Respondents

Out of the 22 graduates contacted, 17 filled out the survey. Of the 17 respondents, 10 graduated within 5 years, and seven graduated more than 5 years before the date when the survey was sent out.

Regarding CAMPEP degrees that respondents held when starting the residency program, 10 had no degree from a CAMPEP‐accredited program, while seven held a M.S., Ph.D., or certificate from a CAMPEP‐accredited program.

### 3.2 Factors considered at the time of application to the residency

**FIGURE 1:**
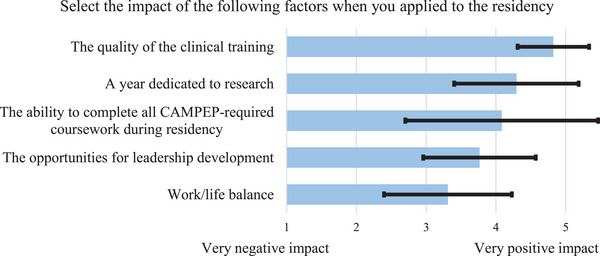
Average (length of bar) and standard deviation (error bar) over all respondents of rankings of the impact of various factors when applying to residency programs. Factors were sorted from highest to lowest average rankings.

The impact of different aspects of the residency program on respondents' decision to apply is shown in Figure 1. Respondents ranked the impact of each factor on a scale of 1–5, with 1 corresponding to a “very negative impact” and 5 to a “very positive impact”. Responses were averaged and sorted in order of highest to lowest ranking. The quality of the clinical training received the highest average score (average score of 4.8, standard deviation of 0.5), and “A year dedicated to research” receive the second highest score (average = 4.3, standard deviation = 0.9). Work/life balance had the lowest average score (average = 3.3, standard deviation = 0.9). The largest standard deviation (1.4) was observed for the factor “the ability to complete all CAMPEP‐required coursework during residency”, indicating large variability of responses in this category. The standard deviations for other responses were ≤ 0.9, indicating closer agreement between the respondents.

**FIGURE 2:**
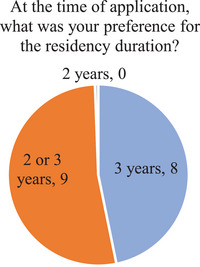
Distribution of responses to question about graduates’ preference for residency duration, in retrospect, at the time of application to residency programs.

Figure 2 shows the distribution of preferences for residency duration at the time of application. The responses were split almost equally between those with a preference for a 3‐year program and those considering both 2‐ and 3‐year programs. No respondents had a preference of a 2‐year program at the time of application.

### 3.3 Career and life aspects affected by graduating from a 3‐year program

**FIGURE 3:**
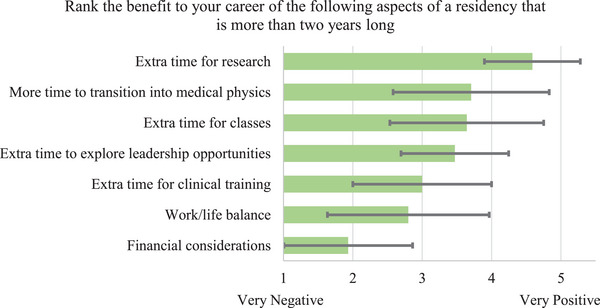
Average (length of bar) and standard deviation (error bar) of rankings of the benefit to respondents' careers of several aspects of the 3‐year residency program. Factors were sorted from highest to lowest average rankings.

Responses to the question about the benefit to the respondent's career of several aspects of a residency program that is more than 2 years long are summarized in Figure 3. “Extra time for research” received the highest score (average = 4.6, standard deviation = 0.7) and “Financial considerations” received the lowest score (average = 1.9, standard deviation = 0.9). “Work/life balance” received the second lowest score (average = 2.8, standard deviation = 1.2) on this question.

**FIGURE 4:**
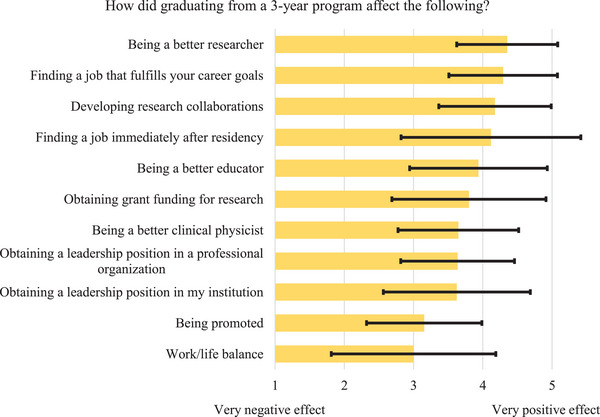
Average (length of bar) and standard deviation (error bar) of rankings of the effect of graduating from a 3‐year program on various aspects of respondents' careers. Factors were sorted from highest to lowest average rankings.

In Figure 4, the average rankings for the impact of graduating from a 3‐year program on several aspects of the respondent's post‐residency career are shown. “Being a better researcher” received the highest average ranking (average = 4.4, standard deviation = 0.7) and “Work/life balance” received the lowest average ranking (average = 3.0, standard deviation = 1.2) among the respondents.

Table 1 summarizes responses to the questions related to the graduates' current positions. Ten out of 17 respondents reported having at least one leadership position (clinical, research or in residency) and some reported holding several leadership positions. The majority of respondents (53%) are satisfied with the way their time is split between different services while 24% reported not having enough time for everything and reducing their time for teaching and research. The number of years of experience from residency given in salary negotiations varied between the respondents, with 2 years reported by 35% of responders while 52% responders reported that “this never came up”.

**TABLE 1:** Responses to survey questions regarding respondent's career after residency.

	Number of respondents	% of respondents
A. Select all of the following that apply to you:		
I hold a clinical research position	6	35%
I run a research group	5	29%
I hold a leadership position in a residency program	4	24%
None of the above	7	41%
B. In your current position, are you satisfied with the way your time is divided between clinical, research, teaching, and other academic pursuits?		
Yes	9	53%
Not enough time for everything, had to reduce teaching and research	4	24%
N/A or empty	4	24%
C. How many years of experience from residency were you given credit for in salary negotiations after residency?		
N/A, this never came up	9	53%
0 years	1	6%
2 years	6	35%
3 years	1	6%

*Note*: Note that the percentages for question A add to > 100% as several respondents hold multiple types of leadership positions.

### 3.4 Opinions on the residency duration

**FIGURE 5:**
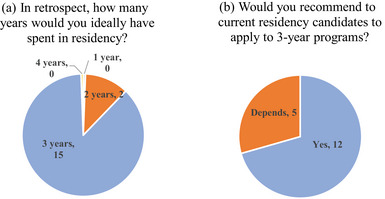
Distribution of responses to questions about perception of the following: (a) ideal number of years in residency and (b) whether respondents would recommend 3‐year programs to new applicants.

The distribution of the number of years, in retrospect, they would ideally have spent in residency is shown in Figure 5a. Three years was selected as the preference by the majority of respondents (15/17) and 2 years was selected by only two respondents. No respondents had a preference for either 1‐ or 4‐year residency duration. Figure 5b shows the distribution of responses of the graduates whether they would recommend to current residency applicants to apply to 3‐year programs. Most of the respondents (12/17) replied “yes” while (5/17) said “it depends”.

When asked whether they agreed with CAMPEP that medical physics residencies should require 2 full years of clinical training, all 17 respondents said yes, that this was the right amount of clinical training during residency.

**FIGURE 6:**
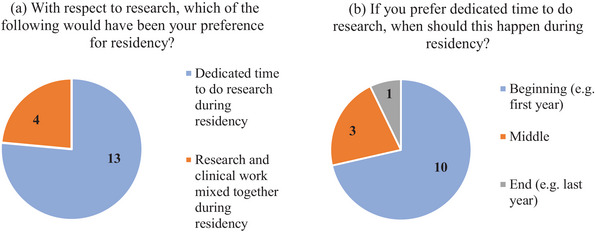
Distribution of response to questions about (a) dedicated time versus mixed time for research and (b) timing of the research component during residency.

The survey included questions asking whether respondents preferred dedicated time to do research, and if so, when this dedicated time should be during the residency. The responses to these questions are shown in Figure 6. The majority of respondents (13/17) preferred to have dedicated time to do research (Figure 6a). The majority (10/17) respondents also expressed their preference toward research in the beginning of the residency while 3/17 and 1/17 had their preference toward research in the middle and end of the residency, respectively (Figure 6b).

### 3.5 Research productivity during residency

**FIGURE 7:**
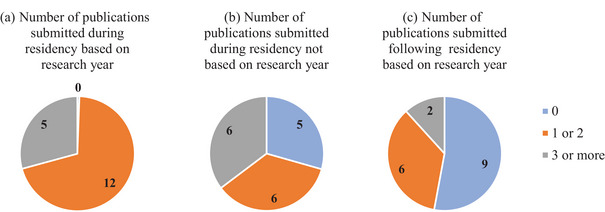
The number of publications respondents recalled submitting in surveyed categories: (a) during residency, based on their dedicated research year, (b) during residency, not based on the dedicated research year, (c) after residency, based on the dedicated research year.

Respondents reported the number of papers submitted during residency, both related and unrelated to the research performed during the dedicated research year, as well as the number of papers submitted after residency related to their research year. The results are presented graphically in Figure 7. All respondents submitted at least one publication based on the research year (Figure 7a) and eight respondents submitted additional publications based on the dedicated research year after graduating from residency (Figure 7c). Twelve respondents also submitted publications during residency not based on their research year (Figure 7b).

### 3.6 Free text comments

Out of 22 respondents, seven provided free‐text comments. All comments were positive and highlighted the following aspects: (1) a 3‐year program may not benefit every candidate; (2) a 3‐year program is important for those coming from backgrounds outside of medical physics; (3) the research component is an important part of the residency training; (4) 3‐year program provides opportunities for research and developing leadership skills. Additionally, interest was experessed in opportunities for grant applications during residency.

## 4 DISCUSSION

In this report we described a survey of graduates of a 3‐year medical physics residency program with a year dedicated to research. The survey was designed to evaluate graduates' perception of their experience, and whether this 3‐year model for a residency was beneficial to their careers. Seventeen graduates of the program filled out the survey, corresponding to a high response rate of 77%.

Overall, the perception of the 3‐year residency duration was positive among those surveyed. Although survey respondents included similar numbers of those who were initially interested in a 3‐year program (8/17) as those who were considering both 2‐ and 3‐year programs (9/17), the majority (15/17) respondents reported that in retrospect, they would ideally have spent 3 years in the residency. No respondent would have preferred a 4‐year program duration. Most respondents (12/17) indicated that they would recommend a 3‐year residency to candidates currently applying, though several (5/17) mentioned that this recommendation would depend on the candidates' background and future goals. Research was mentioned as one of the leading factors for training in a 3‐year program. The graduates assigned high rankings to “Extra time for research” in their responses to the question about the benefits of a 3‐year program and “Being a better researcher” on a question about effects of graduating from a 3‐year program.

In this report, research productivity was assessed by the number of publications submitted during and after residency. The responses to questions included in the survey to evaluate the effect of the dedicated research year on graduates' research productivity indicated clear benefits of a protected year of research. While productivity varied, all respondents submitted at least one publication during residency based on their dedicated research year, and 5/17 submitted 3 or more publications. The fact that the majority of residents (70%) in a 3‐year residency produced 1−2 publications may indicate that at least a year of research is needed to allow submission of a publication.

The survey also tested the role of research continuity on the number of publications produced by the residents. While many respondents (11/17) were able to submit papers based on work outside of the dedicated research year, a significant fraction (6/17) did not submit other papers during residency, indicating that the research year was an important opportunity to achieve research productivity during residency and future engagement in medical physics research. In this survey, 8/17 respondents continued their research collaboration from the dedicated year in some fashion, submitting additional papers related to this research after graduation. Respondents ranked “Being a better researcher” and “Developing research collaborations” as two of the most positive effects of graduation from a 3‐year program. These results indicate that extra year of research is particularly beneficial to those who are interested in pursuing research in their future career.

In addition to the residency duration, another important consideration when including research in the residency training is the timing of the research component within the residency program. While doing research in the beginning of the residency may help the incoming resident get acclimated to a new field and perhaps complete the didactic component of their training, it may put the resident at a disadvantage in being able to understand the clinical impact of their work if they do not enter the residency with medical physics background. We therefore asked the graduates to report on their preferences with respect to timing research in their training. The majority of respondents preferred to have dedicated time during residency instead of mixed with clinical work and expressed their preference to have research in the beginning of the residency. While there were other responses, dedicating the last year of residency to research was only preferred by one respondent.

We also aimed to evaluate if the potential preference for research during the residency comes at the cost of high‐quality clinical training. To test this hypothesis the survey included questions about graduates' perception of clinical training at the time of application and after graduation. While the graduates of our program had a clear interest in research, the results of the survey indicate that the quality of clinical training was a high priority for the incoming residents. On average, the graduates reported the quality of clinical training to have very positive impact on their decision to apply to the residency. The respondents provided a high score (3.6) for an option of “Being a better clinical physicist” as an effect of graduating from a 3‐year program, and 100% responders said “yes” to a question if they agree that 2 full years should be required for clinical training. Many graduates also reported holding clinical leadership positions after graduation. These results again indicate that extra time is required in a residency program that is interested in providing research opportunities to the resident while maintaining high quality of clinical training and preparing future graduates for success in all aspects of their future medical physics career.

Since a sizable fraction of residents in the 3‐year program studied in this report enter the residency program without a CAMPEP‐accredited degree, it was important to evaluate if the graduates' preference for the residency duration was affected by their CAMPEP degree status. In order to qualify for the ABR board certification process, they must complete all required courses for a CAMPEP‐accredited certificate in medical physics during the residency program. Only two of these courses may be taken during years dedicated to clinical training. Therefore, residents in this category must take most of the certificate courses during the dedicated research year. Among the survey respondents without prior CAMPEP‐accredited degree (10/17), the ability to complete all CAMPEP‐required coursework during the residency was an important factor in applying to the residency program (average = 4.5, standard deviation = 1.1), and the benefit of the residency in providing extra time to complete classes was scored at an average of 3.9 in this group of respondents. While an opportunity to satisfy the didactic requirements is an attractive option for those without prior CAMPEP degree, this group of applicants is fairly small. When evaluated for a wider group of applicants, the opportunity to take didactic courses might not be viewed as a sufficiently favorable factor for extending the residency. Nevertheless, our results suggest that careful consideration should be given when extending the duration of the residency to 3 years and options for fulfilling didactic course requirements by the residents should be factored in when designing a 3‐year program.

In addition to evaluating the benefits of a 3‐year residency, it was equally important to review the potential downsides of extending the duration of residency training. Based on the survey responses regarding career benefits of a 3‐year residency, the lowest‐scoring aspect was financial impact. The financial impact of a 3rd year of residency may be two‐fold: an additional year at a resident (or post‐doctoral researcher) salary; and falling behind a year on the salary scale since this research year may not be considered a year of experience. While the first factor is self‐evident, it is difficult to evaluate the latter effect in this survey, as the majority of respondents (9/17) say the number of years of experience never came up when negotiating their salary after residency, but only one respondent was given credit for 3 years of residency experience.

Another factor that received a low score was work‐life balance. It received the lowest average score in two questions, the question “How did graduating from a 3‐year program affect the following?” and in the question “Rank the benefit to your career of the following aspects of a residency that is more than 2 years long”. These results are not surprising since residents typically work long hours, both during their dedicated research year and during clinical training years, making it difficult to achieve good work‐life balance. This dynamic may continue after residency as well, as graduates from a 3‐year program with research are likely to continue to seek research opportunities, potentially resulting in more time spent working.

As medical physics educational requirements continue to develop, it is likely that efforts will be made to formalize and standardize the research component of the residency curriculum. Our report will provide useful information about graduates' preferences for the duration and composition of the research module in the residency training. To the best of our knowledge, this is the only survey that aimed to gather perceptions of the benefits and drawback of extending the residency to incorporate research component in its curriculum, and as such it lays a foundation for future discussions on this topic. This report has also limitations: it only included graduates of a specific 3‐year medical physics residency program with a dedicated research year. Therefore, the respondents represent a group of physicists who prioritized research during residency whose perceptions likely do not reflect those of a broader group of medical physics residency graduates. In addition, specifics of research year management may be different in other residency programs which also might influence the resident's perception. Further, their perceptions likely indicate some respondent bias, with favorable views of their own residency experience. On the other hand, this group is uniquely situated to evaluate the experience of having completed a 3‐year program and moved on to career positions, and we believe their perceptions provide valuable insight into advantages and disadvantages of this type of residency program. Conducting broader studies including graduates from other 2‐ and 3‐year programs would be beneficial to evaluate the perspectives of other residency graduates. This would help to expand our understanding of best pathways for offering a balanced combination of research and clinical training to future medical physicist.

## 5 CONCLUSION

The optimal duration of the medical physics residency depends on the goals and career objectives of the residents. Two years may be an optimal duration for clinical training as perceived by residency graduates. For candidates interested in a career with a substantial research component, a 3‐year program appears to be a good option that is evaluated positively by graduates of such a program. Four‐year residency duration is not viewed favorably by the graduates.

## AUTHOR CONTRIBUTIONS

Martina Hurwitz and Yulia Lyatskaya drafted the survey, performed the data analysis, and drafted the manuscript. Brian Winey, Piotr Zygmanski, W.S. Kiger III, and David P. Gierga contributed substantially to analysis and interpretation of the work, as well as contributing to the manuscript draft and its revision.

## CONFLICT OF INTEREST STATEMENT

The authors declare no conflicts of interest.
